# A *Trichinella britovi* outbreak in the Northern Alps of France: investigation by a local survey network[Fn FN1]

**DOI:** 10.1051/parasite/2023017

**Published:** 2023-05-12

**Authors:** Martin Peju, Bérangère Granier, Cécile Garnaud, Marie-Pierre Brenier-Pinchart, Isabelle Vallée, Aurélie Chevillot, Charlotte Mérel, Fanny Chereau, Muriel Deher, Olivier Rogeaux, Hélène Yera

**Affiliations:** 1 Infectious diseases unit, Centre Hospitalier Métropole Savoie Place Lucien Biset 73000 Chambéry France; 2 General Practitioner 38570 Crêts en Belledonne France; 3 Institut de Biologie et de Pathologie, Département des Agents Infectieux, Parasitologie – Mycologie, Centre Hospitalier Universitaire Grenoble – Alpes 38000 France; 4 ANSES, École Nationale Vétérinaire d’Alfort, INRAE, UMR BIPAR, Laboratoire Santé Animale 97400 Maisons-Alfort France; 5 Direction Départementale de la Protection des Populations – Service Qualité et Sécurité des Aliments 38000 Grenoble France; 6 French National Public Health Agency, Department of Infectious Diseases 94410 Saint-Maurice France; 7 Agence Régionale de Santé, Pôle Santé Publique 69003 Lyon France; 8 Reference Laboratory for Human Trichinellosis, Hôpital Cochin, APHP Centre, Université de Paris Cité 75014 Paris France

**Keywords:** *Trichinella britovi*, France, Outbreak

## Abstract

We describe a small family outbreak of trichinellosis caused by the consumption of raw ham from a wild boar (*Sus scrofa*) hunted in the northern Alps of France in February 2022. Out of the six people, aged 3–69 years, who consumed the meat, three were confirmed cases, and three were suspected cases. Eosinophilia detected in four people was the hallmark that drove the diagnosis. Three patients presented with myalgia, two with intense and prolonged chest pain, and one with elevated troponin. One patient presented with dermographism during treatment. Anti-*Trichinella* IgG were detected in three symptomatic individuals after about ten weeks. One patient had negative serology and no symptoms, but was on long-term corticosteroid therapy. *Trichinella britovi* larvae (8.3 larvae/g) were detected in the wild boar meat remnants. Trichinellosis is rare in France, but this family outbreak is reminiscent of the circulation of this pathogen in wild animals, highlighting the need to inform hunters about the risk of infection linked to the consumption of raw meat of game animals, and about the need for veterinary inspection of game meat. The consumption of raw meat outside controlled circuits is a practice not devoid of risks, which justifies raising the awareness of hunters, doctors, and medical biologists.

## Abstract (fr)

Nous décrivons une épidémie familiale de trichinellose causée par la consommation de jambon cru d’un sanglier (*Sus scrofa*) chassé dans le nord des Alpes françaises en février 2022. Sur les six personnes âgées de 3 à 69 ans qui ont consommé la viande, trois étaient des cas confirmés, et trois étaient des cas suspects. L’éosinophilie détectée chez quatre personnes a permis d’évoquer le diagnostic. Trois patients présentaient des myalgies, et deux des douleurs thoraciques intenses et prolongées dont un avec une troponine élevée. Un patient a présenté un dermographisme pendant le traitement. Des IgG anti-*Trichinella* ont été détectées chez trois individus symptomatiques après environ dix semaines. Un des patients avait une sérologie négative et aucun symptôme mais était sous corticothérapie au long cours. Des larves de *Trichinella britovi* (8,3 larves/g) ont été détectées dans les restes du jambon de sanglier incriminé. La trichinellose est rare en France, mais cette épidémie familiale rappelle la circulation de cet agent pathogène chez les animaux sauvages, qui nécessite d’informer les chasseurs sur les risques d’infections liés à la consommation de viande crue de gibier, et de préconiser un contrôle vétérinaire des viandes de gibier. La consommation de viande crue en dehors des circuits contrôlés est une pratique non dénuée de risques, qui justifie une sensibilisation des chasseurs, médecins et biologistes médicaux.

## Introduction

Trichinellosis is a foodborne parasitic disease transmitted by consuming infected raw meat [14]. The *Trichinella britovi* species infects wild animals of Europe, North and West Africa, and Southwest Asia. The microscopic larvae, present in the muscle cells of infected meat, are released by stomach digestion and mature within 48 h in the small intestine. Five to 15 days after infection, the females produce thousands of larvae after mating. The adsults die in fewer than four weeks. The newborn larvae (NBL) migrate into the general circulation and then move into the muscle fibers. There, the NBL divert the metabolism of the host cell, which becomes a nurse cell. They encyst three weeks after contamination and survive from few weeks up to several years, depending on the parasite and host species [17, 27]. As the NBL leave the intestine to reach the bloodstream and muscles, the main symptoms are myalgia and eosinophilia. The heart may be involved with life-threatening myocarditis and rhythm abnormalities, although larvae do not penetrate the myocardial muscle cells [22]. Central nervous system damage is sometimes described [31]. All carcasses of *Trichinella* spp. susceptible animals whose meat is intended for market must be tested according to EU and French regulations [10, 20, 21]. This infection is thus easily avoided by veterinary inspections at the slaughterhouses for pigs or wildlife processing centers for wild boar meat (*Sus scrofa*). All trichinellosis infections observed in France since 1998 are thus only related to the consumption of uncontrolled meat [11]. As trichinellosis infection is rare, it can be overlooked. We describe here the first cases of human infection by *Trichinella britovi* in the Northern Alps of France and the role of a local survey network in the initial investigations.

## Case report

**Figure 1:**
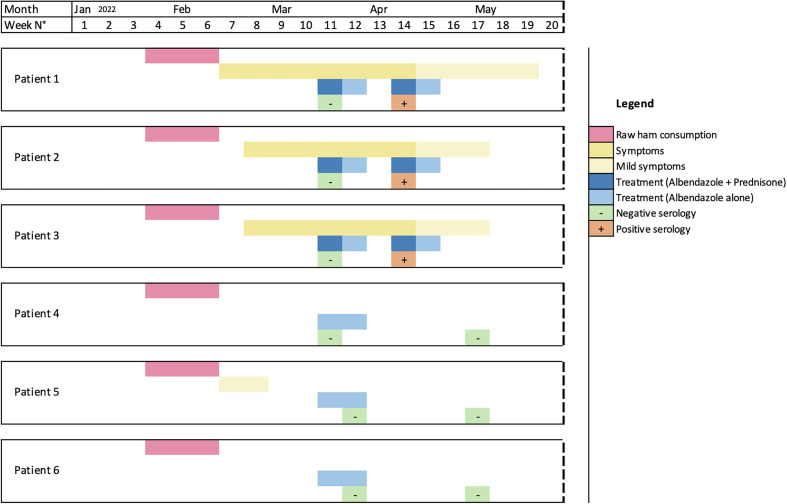
Estimated timeline of the outbreak.

We describe an outbreak of trichinellosis, which involved a six-person family belonging to the same household (four adults and two children), some of whom experienced signs and symptoms of *Trichinella* infection after eating salted raw wild boar meat that they had hunted a few months prior. Trichinellosis was suspected as three cases of muscle pain with elevated creatinine kinase, and eosinophilia occurred in the same family of hunters. This disorder was quickly considered because the family doctor was able to easily seek advice from infectious disease specialists via a local network involving professionals from the hospital and health authorities. The food causing the infection was rapidly identified as meat from a wild boar hunted in mid-December 2021 near the town of Allevard in the Belledonne massif of the French Alps. A hind leg of the animal had been salted and put out to dry to make raw ham. The family started consuming the ham at the end of January 2022, and symptoms began around mid-February 2022 (see Table 1; Fig. 1). Using artificial digestion, 8.32 larvae/g of meat were detected in the ham [10]. The larvae were identified as *T. britovi* after DNA extraction and multiplex PCR [18]. All patients were treated with albendazole 15 mg/kg/day for 14 days, while prednisone 1 mg/kg/day for one week was used in cases 1, 2 and 3, as their symptoms were pronounced. Asymptomatic family members were also treated because the whole family had been consuming the infected ham over a long and imprecise period. Serology performed in March was negative in all patients (commercial tests used – ELISA: Novalisa *Trichinella spiralis* IgG, Novatec, Orléans, France; for Western Blot: LD BIO Diagnostics, Lyon, France). However, the diagnosis was retained because of the presence of larvae in the consumed meat, with some of the patients having compatible symptoms and eosinophilia. The second serology performed in April in patients 1, 2 and 3 was positive by ELISA and Western Blot. These three patients were subjected to a second cycle of treatment of 14 days of albendazole and 7 days of prednisone because of persistent or recurring symptoms despite the first line of treatment. The second serology remained negative for patients 4, 5 and 6.

**Table 1:** Summary of the medical records of the six patients involved. Bold: abnormal results.

	Patient 1	Patient 2	Patient 3	Patient 4	Patient 5	Patient 6
Sex	Male	Female	Female	Male	Female	Female
Age	36	62	30	69	8	3
Medical history	Heavy active smoking.	Mitochondrial myopathy, pulmonary emphysema	None	Rheumatoid arthritis, under prednisone treatment 5 mg/day	None	None
Consumption of infected meat	From the end of January, 2022					
Date of first symptoms	Mid-February	Mid-February	Mid-February	NA	Mid-February	NA
Signs and symptoms	Asthenia, dyspnea, chest pain, low fever	Myalgia, asthenia, eyelid edema, headache, low fever	Myalgia, dizziness, nausea, low fever, dermographism	None	Non-specific stomach aches	None
Maximum eosinophil count (G/L) (*N* < 0.5)	**3.00**	**1.37**	**1.81**	0.33	**1.20**	0.3
Maximum creatine phosphokinase (U/L) (*N* < 168)	**300**	98	**250**	**320**	**222**	**334**
Maximum lactate dehydrogenase (U/L) (*N* < 220)	**339**	**286**	**239**	**234**	NA	NA
Maximum troponin (ng/L) (*N* < 14)	51	18	NA	NA	NA	NA
Electrocardiogram	Normal	Normal	NA	NA	NA	NA
Echocardiography	Normal	Normal	NA	NA	NA	NA
First serology						
ELISA	Negative	Negative	Negative	Negative	Negative	Negative
Western Blot	Negative	Negative	Negative	Negative	Negative	Negative
Second serology						
ELISA	**Positive**	**Positive**	**Positive**	Negative	Negative	Negative
Western Blot	**Positive**	**Positive**	**Positive**	Negative	Negative	Negative
Status	**Confirmed case**	**Confirmed case**	**Confirmed case**	Suspected case	Suspected case	Suspected case
Treatment	Albendazole 15 mg/kg/day, 14 days; prednisone 1 mg/kg/day, 7 days			Albendazole 15 mg/kg/day, 14 days		
Follow-up	Improvement during treatment, then relapse of the symptoms.			Still no symptoms so far		
	Improvement after the second course of albendazole and prednisone					

The patients have been informed of the use of their medical data and of their right to refuse.

Patient 1 was a 36-year-old man with no history other than heavy active smoking. He presented with strong asthenia, moderate fever around 38 °C, and dyspnea in mid-February. His condition worsened with the onset of intense chest pain. ECG and echocardiography were normal, but troponin was elevated at 50 ng/L (*N* < 13 ng/L), indicating ongoing myocardial damage. The eosinophil count was elevated, at 3.02 G/L. The symptoms improved under treatment (albendazole plus prednisone), but asthenia persisted for weeks. He received a second cycle of treatment for 14 days. Eosinophil level and troponin returned rapidly to normal from the first days of treatment.

Patient 2 was a 62-year-old woman, monitored for mitochondrial myopathy under levocarnitine and post-smoking pulmonary emphysema. She had signs and symptoms from mid-February: diffuse and intense myalgia, with significant asthenia, eyelid edema, moderate fever around 38 °C, headaches, and a feeling of “brain fog,” all progressing to chronic asthenia with significant myalgia and chest pain for several weeks. The assessment found eosinophilia at 1.37 G/L, troponin was normal, and ECG and echocardiography were unremarkable. The symptoms improved under treatment (albendazole plus prednisone) but recurred when treatment was stopped. She was consequently subjected to a second cycle of treatment of 14 days. The eosinophil level returned to normal from the first days of treatment.

Patient 3 was a 30-year-old woman with no medical history. At the end of February, she presented with nausea, myalgia, dizziness, and moderate transient fever around 38 °C that improved spontaneously in a few days. However, a complete blood count found hypereosinophilia at 1.81 G/L. She was treated with albendazole and prednisone from mid-March along with other family members identified as potential cases of trichinellosis. The drugs were well tolerated, but she complained of a rash with intense pruritus at the end of treatment. This eruption was caused by physical stimulation of the skin and corresponded to frank dermographism. Transaminases were normal. The eosinophil level returned rapidly to normal.

Patient 4 was a 69-year-old man with rheumatoid arthritis on prednisone 5 mg/day. He did not report any signs or symptoms. Clinical analyses only detected a slight elevation of CPK (320 U/L) and LDH (234 U/L), but the eosinophil count was normal. Early and late serology remained negative by screening and confirmatory tests (ELISA and Western blot). He was treated with albendazole only.

Patient 5 was an 8-year-old girl who did not complain of any symptoms apart from non-specific stomach aches several weeks after ingesting the ham, but she nonetheless underwent a work-up which revealed eosinophilia at 1.2 G/L and a moderate elevation of CPK (222 U/L). She was treated at the same time as her family with albendazole only. Early and late serology remained negative in screening and confirmatory tests (ELISA and Western blot).

Patient 6 was a 3-year-old girl with no symptoms in whom only a moderate elevation of CPK was found (334 UI/L). The eosinophil count was normal. She was treated with albendazole only. Early and late serology remained negative in screening and confirmatory tests (ELISA and Western blot).

Based on case definitions from the World Health Organization guidelines [8], patients 1 to 3 were confirmed cases, and patients 4 to 6 were suspected cases.

## Discussion

**Figure 2:**
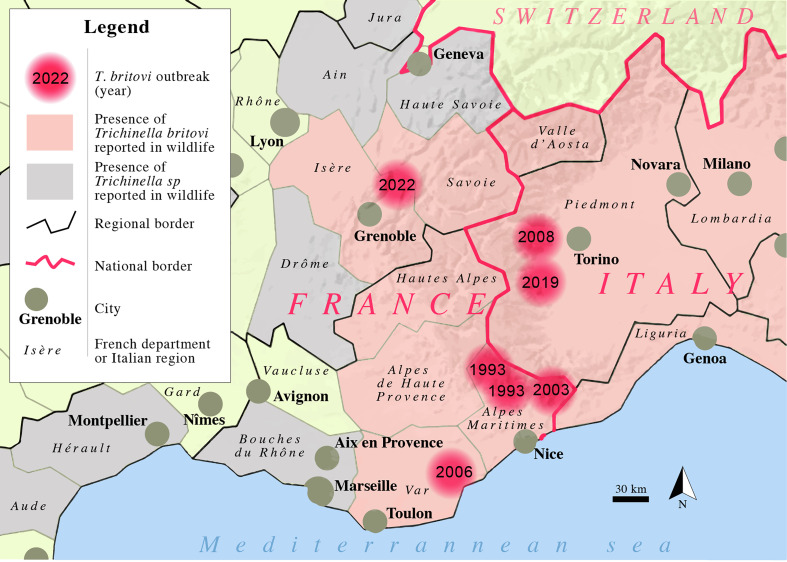
Map of the Alps with location of recent outbreaks of trichinellosis due to *Trichinella* *britovi*, and presence of *Trichinella britovi* or *Trichinella* sp in wildlife. Outbreaks of trichinellosis due to other species of *Trichinella* sp. are not represented.

The study describes a small family outbreak of trichinellosis caused by the consumption of raw ham made with wild boar meat. In the French Alps of the Savoie, Isère, and Haute-Alpes Departments, *T. britovi* was detected in six wolves (*Canis lupus*) and a wild fox (*Vulpes vulpes*) between 2007 and 2013 [35]. Wild boars can easily acquire the infection by feeding on infected carcasses of wild animals left by hunters in the field or killed on the roads, and, therefore, represent a health risk if not properly inspected by veterinarians [36]. The presence of *T. britovi* in wild foxes and/or wild boars has been reported in the past, in all regions of Italy and departments of France adjoining the Alps [25, 28]. *Trichinella britovi* was reported in wild foxes of the Var department, and seroprevalences to *Trichinella* sp. greater than 10% were observed in wild boars in several departments of the south-east of France (see Fig. 2) [1, 15]. This is the first description of human trichinellosis in the Isère Department and of *T. britovi* in humans in the Northern Alps of France. Human trichinellosis due to *T. britovi* has previously been reported in three outbreaks in the Alpes Maritimes department and one in the Var department (see Fig. 2) [3, 6, 12, 13]. The source of infection was the consumption of raw wild boar ham in 1993, and undercooked frozen (at –35 °C for seven days) wild boar meat in 2003. A fourth outbreak occurred in the Alpes Maritimes department, but it has no epidemiological link with the other three as it was caused by the consumption of raw pork sausages (*figatelli*) prepared in Corsica in 2016 [32]. Two other outbreaks (up to 35 confirmed cases) due to *T. britovi*, caused by the consumption of wild boar meat, occurred in the Piedmont region (Western Alps of Italy) in 2008 and 2019 [30, 34]. These outbreaks highlight the risk of acquiring trichinellosis from wild boar meat in the Alps.

In France, eosinophilia is more frequently associated with iatrogenic reactions or allergies, and if parasitic diseases are found, they are more probably the result of recent travel abroad [24]. Indigenous parasitic diseases are rare, even though it is known that wild animals of France harbor *Trichinella* spp [1, 6, 15, 19, 29, 35]. Apart from an imported outbreak with nine cases in 2017 (due to the consumption of pork from Serbia), there have only been two indigenous outbreak in the last ten years, with five patients involved [11]. Since this disease occurs seldom, general practitioners or specialists can overlook it, and diagnosis may be delayed or missed. In this case, it is interesting that the first doctor consulted reacted quickly to eosinophilia in several patients, using a local network (called InfMed) to obtain specialized advice. The InfMed exchange forum was created in 2008 at the departmental level, on the initiative of private and hospital doctors (infectiologists and pediatricians), to facilitate communication between general practitioners and specialists on infection-related topics [16]. Each participant can ask questions and receive alerts, and access protocols or opinions on infectious diseases or vaccination. This made early diagnosis possible, and public health authorities, that are also invested in this network, were alerted. The SARS-CoV-2 pandemic marked a sharp increase in participating doctors, demonstrating the value of discussion and information in the profession.

Notably, none of the affected patients knew of the risks of consuming raw wild boar meat, even though some were hunters. Meat inspection for *Trichinella* spp. larvae is mandatory for any commercialized pork and wild boar meat in France (under EU regulation) and is well supervised in France since veterinary controls are carried out in slaughterhouses or wild game meat processing centers. Wild boar meat inspection is strongly recommended but not mandatory for personal consumption. Hunters can collect samples for examination by veterinary services. As a last resort, if hunters cannot have the meat analyzed, then cooking the meat will kill the parasite. Freezing is insufficient to ensure the inactivation of the larvae in game meat. Awareness of exposure to the risk of trichinellosis is probably not high enough for hunters since it is a rare event.

Human infection by *T. britovi* is generally not severe due to the low fertility of female worms [5]. In the pig model, *T. britovi* has less infectivity and immunogenicity and a shorter larva survival period in muscles than *T. spiralis* [27]. The lower infectivity could explain the attack rate of 50% observed in this report (three confirmed and three suspected cases out of six contacts). This is consistent with the literature [5]. Not surprisingly, two of the three patients considered suspected cases of trichinellosis were children. Myalgia and complications of trichinellosis were detected less frequently in children who also had lower eosinophil counts [23]. Lower infecting doses and a less intense allergic reaction to the larvae invasion could explain the milder clinical presentation. Antibodies may usually be detectable in serum from 15 days after infection and may only appear after eight weeks [4]. The time of appearance for antibodies depends on the infectious dose (the lower the dose, the later the antibodies appear), and the *Trichinella* species [9]. Seroconversion occurs slowly with *T. britovi* compared to *T. spiralis* [27]. This difference in host immune response could explain the delay in seroconversion in our series since the first serology performed from four to six weeks after infection was negative in all patients. Therefore, negative serology associated with highly evocative signs should not exclude the diagnosis during the first weeks or months. Western blot, which can be earlier than ELISA for detecting anti-*Trichinella* IgG in the acute phase [2, 13], should be recommended. Both tests should use excreted/secreted (E/S) antigens of *T. spiralis* [4]. Serology should also be repeated several weeks later. Albendazole is more effective in the early stage of infection than the late stage and fails to be effective to kill muscle encysted larvae [26, 33]. When administrated in infected mice at 37 days post-infection, the reduction percentage in muscle larval load was 35% [7]. Here, the treatment was started more than 40–50 days after infection. Consequently, albendazole had a low effect on encapsulated larvae. The second course of albendazole and prednisone would also have a low effect on muscular larvae but possibly diminish the inflammatory reaction. Interestingly, we noted that case 4, who consumed the infected meat several times, did not present any symptoms or specific IgG more than three months after meat consumption. The only abnormalities were slightly elevated levels of creatine phosphokinase and lactate dehydrogenase. We suggest that the corticoids he was receiving for rheumatoid arthritis prevented the appearance of symptoms and modified the immune response leading to normal blood eosinophil counts and an absence of specific antibodies. Corticosteroids are known to reduce blood eosinophil levels and are used for this purpose in some inflammatory diseases with eosinophilia. Elsewhere, we previously reported one case of trichinellosis that consulted his general practitioner at the outset of the infection for facial paralysis. He received only a high dose of corticosteroids, then had normal blood eosinophil counts and muscle enzymes and a delay in the appearance of specific antibodies (only in Western blot) [2].

## Conclusion

The rarity of the disease, lack of public awareness of this parasitosis, and the fact that food safety is taken for granted in France, probably contributed to making this series of cases possible, but the circulation of *Trichinella britovi* is now well documented in the Alps. Although parasitic diseases and expertise in this field are rare in France, fluid collaboration between practitioners and specialists facilitates rapid reaction in the presence of signs like eosinophilia. Hunters are highly exposed and must be informed of the risks of wild boar meat consumption. Serological tests may be negative during the first weeks of infection and should not delay the diagnosis and treatment in the presence of evocative signs and symptoms of trichinellosis.
